# Anticipation in familial lattice corneal dystrophy type I with R124C mutation in the *TGFBI* (*BIGH3*) gene

**Published:** 2008-05-07

**Authors:** Pablo Romero, Marlene Vogel, Jose-Manuel Diaz, Maria-Patricia Romero, Luisa Herrera

**Affiliations:** 1 Depto de Oftalmología, Hospital Clínico José Joaquin Aguirre, Universidad de Chile, Santiago, Chile; 2 Programa de Genética Humana, ICBM, Facultad de Medicina, Universidad de Chile, Santiago, Chile

## Abstract

**Purpose:**

To report the clinical, ophthalmic, and genetic characteristics for lattice corneal dystrophy type I (LCDI) in a Chilean family.

**Methods:**

Six affected family members were examined clinically including visual acuity, color cornea photography, applanation tonography, and fundoscopy. Genomic DNA was extracted from peripheral leukocytes from six affected and three unaffected members of a family with lattice corneal dystrophy type I. Exon 4 of the *transforming growth factor-induced* gene (*TGFBI*) was screened for the most frequent mutation, R124C, in the proband by sequencing. We also designed a rapid polymerase chain reaction-restriction fragment length polymorphism (PCR-RFLP) method to analyze the same mutation, amplifying exon 4 and digesting with PstI restriction enzyme. Using this strategy, we analyzed the mutation in six affected and three healthy family members.

**Results:**

Three generations of family members were positively diagnosed with lattice corneal dystrophy. Six participants demonstrated LCD1 in both eyes, most of whom were symmetric. Age at onset of symptoms was variable (3–42 years old). Moreover, in this family, the age of onset of the disease decreased in succeeding generations, which could be interpreted as anticipation. Visual acuity varied from 1.0 to 0.13. Two patients, ages 69 and 44 years old, demonstrated a degree of severity “Bad” according to best-corrected vision and corneal commitment. The exon 4 sequence of *TGFBI* of the proband exhibits the heterozygous single-nucleotide mutation, C417T, leading to amino acid substitution (R124C) in the encoded TGF–induced protein. Using PCR-RFLP, we confirmed the heterozygous mutation in six affected family members and excluded it in three healthy members.

**Conclusions:**

The R124C mutation in *TGFBI* cosegregated with LCD type I in the investigated family. This is the first report of a molecular analysis of LCD type I in Chilean patients. The early onset affected persons in the fourth generation raises the possibility of anticipation.

## Introduction

The corneal dystrophies are a group of genetically determined diseases usually characterized by loss of corneal transparency, which may be caused by a progressive accumulation of abnormal material within the cornea. Lattice corneal dystrophy (LCD) is a distinct clinical entity characterized by the accumulation of amyloid throughout the middle and anterior stroma [1].

Lattice corneal dystrophy type I (LCD1, OMIM 122200), also known as Biber-Haab-Dimmer dystrophy, is inherited as an autosomal dominant trait with variable clinical expression and a high degree of penetration [2]. Generally, the first clinical symptoms become evident in the first or second decade with the appearance of white-grayish opacities, which involve the superficial stromal layer of the cornea. Thereafter, the lesions tend to become larger, aggregate, and extend deeper and toward the periphery.

In 1994, a gene for several corneal dystrophies [3] and the *transforming growth factor-induced* gene (*TGFBI,* also known as *BIGH3*) [4] were mapped to human chromosome 5 (5q31). During the same year, it was also discovered that the protein product of the *TGFBI* gene is expressed in the cornea [5]. Three years later, Munier et al. [6] reported that these disorders are caused by mutations in *TGFBI*. Mutations in the same gene also cause granular corneal dystrophies (GCD; reviewed by Kannabiran and Klintworth [7]). *TGFBI* encodes for a 68 kDa extracellular matrix protein [4].

So far, few mutations have been described in this gene as causative of LCD type I. The mutation list includes R124C, V505D, L518P, V539D, A546D, P551Q, L569R, H572R, and V625D [7-9]. The mutation C>T at the nucleotide position 417 at codon 124 (R124C) in exon 4 is the most frequent throughout the world [6,10-21].

Considering that there are no genetic studies on lattice corneal dystrophy in our country, we determined the clinical, ophthalmic, and genetic characteristics in a Chilean family having this disease. Our results suggest anticipation in the disease. We report for the first time the presence of the *TGFBI* mutation segregating with lattice corneal dystrophy type I in Chilean patients. The molecular basis of anticipation in LCDI, which is not mediated by trinucleotide repeat expansions in the *BIGH3*, remains elusive.

## Methods

### Patients

**Figure 1:**
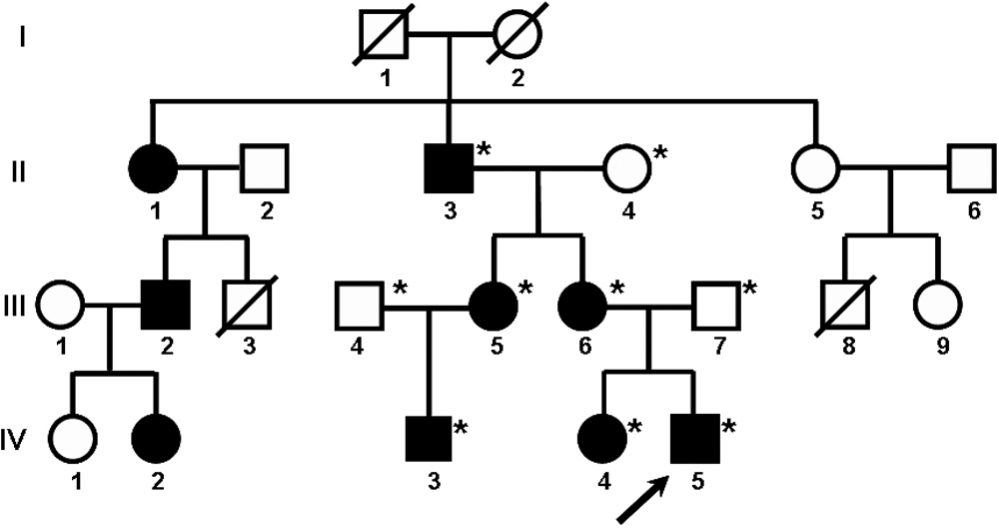
Pedigree showing a four-generation family affected by lattice corneal dystrophy type I. Autosomal dominant transmission of the disease is evident. Circles represent females, squares represent males and a slash mark through the square or circle indicates deceased. The arrow indicates the proband, and the filled symbols indicate affected individuals. Asterisks indicate members of the family who underwent clinical examination and molecular analyses.

The present study was approved by the Ethics Committee of the Clinical Hospital University of Chile and adhered to the tenets of the Declaration of Helsinki. The pedigree of the four-generation family was delineated (Figure 1). After obtaining informed consent, nine members from three consecutive generations of a Chilean family with lattice corneal dystrophy were enrolled, six affected and three unaffected. The proband was initially examined because of ocular pain and redness. The LCD type I diagnosis was made on the basis of clinical examination. The nine members of the family, affected and not affected, were examined over two years. The family history suggests Spanish origin.

### Clinical evaluations

An extensive clinical history of participating members of the family was developed and particular attention was paid to the age of onset, initial signs and symptoms, and corneal structure. Clinical examination included best corrected visual acuity according to the best line of Snellen acuity, slit lamp biomicroscopy, color cornea photography, applanation tonography, and dilated fundus examination. Autorefractometry measurement and keratometry were performed (Topcon RM-A7000, Tokyo, Japan). Stereopsis was quantitatively assessed with the Titmus Fly Stereo Test (Stereo Optical Co., Inc., Chicago, IL) [22]. The lesions were considered to be synchronic if the patients perceived the first symptoms in both eyes with a time difference of less than a month. All individuals with corneal commitment were considered clinically affected. Patients were classified in degree of severity according to best-corrected vision (Table 1) and the number of lesions and corneal commitment. The corneal phenotype of all index patients was assessed by slit lamp biomicroscopy examination and reviewed by an investigator ignorant of the genetic status. Although the patients, II-1, III-2 and IV-2, were not examined in this study, their age of onset were obtained from an interview of patient II-1.

**Table 1:** Clinical features in the affected and unaffected family members.

					**Best-corrected vision**						
**Case**	**Age**	**Gender**	**Status**	**Age of onset**	**OD**	**OS**	**Corneal lesions**	**Synchronic**	**Symmetry**	Degree*	Degree**
II-1	73	F	Affected	37			Yes#				
II-3	69	M	Affected	42	0.13	0.13	Yes	Yes	No	Bad	4
II-4	68	F	Not affected	-	1	1					
III-2	55	M	Affected	17			Yes##				
III-4	42	M	Not affected	-	1	1					
III-5	44	F	Affected	30	0.28	0.13	Yes	No	Yes	Bad	4
III-6	40	F	Affected	28	0.5	0.4	Yes	Yes	Yes	Intermediate	3
III-7	43	M	Not affected	-	1	1					
IV-2	16	F	Affected	5							
IV-3	15	M	Affected	3	1	1	Yes	Yes	Yes	Very good	1
IV-4	15	F	Affected	4	1	1	Yes	Yes	Yes	Very good	1
IV-5	8	M	Affected	4	0.5	0.67	Yes	Yes	No	Good	2

The phenotypic features of nine patients from the family affected by lattice type I are shown. The age of onset of patients II-1, III-2, and IV-2 were obtained from an interview of patient II-1. Corneal phenotypes were assessed by slit lamp examination. The lesions were considered to be synchronic if the patients perceived the first symptoms at most a month apart in each eye. The degree of severity was classified according to best-corrected vision or corneal commitment. The degree of severity according to best-corrected vision was classified as very good (>0.6), good (<0.6 and >0.4), intermediate (<0.4 and >0.33), and bad (<0.33). The degree of severity according to corneal commitment was classified as follows 1) without lesions, 2) subepithelial, white-grayish opacities, 3) refractile lattice lines, and 4) corneal edema. The following abbreviations were used right eye (OD) and left eye (OS). In the table, the asterisk indicates the degree according to best-corrected vision; the double asterisk indicates the degree according to corneal commitment; the sharp (hash mark) indicates a corneal transplant at 43 years of age; and the double sharp indicates a corneal transplant at 55 years of age.

### Molecular analysis

Blood samples were colleted in tubes containing EDTA and total DNA was prepared from peripheral blood lymphocytes [23]. Exon 4 of the proband was amplified using previously described primers [24]. The resulting polymerase chain reaction (PCR) product was purified using the Wizard^®^ Gel purification kit (Promega Corporation, Madison, WI) and sequenced using Big Dye^TM^ sequencing reagents (Applied Biosystems, Foster city, CA), and automated sequencing was performed by capillary electrophoresis on an ABI3700 (Applied Biosystems). To analyze the same mutation in the nine family members, we designed a new simple and rapid detection method based on polymerase chain reaction-restriction fragment length polymorphism analysis (PCR-RFLP). To carry out the PCR-RFLP analyses, genomic DNA from nine family members was amplified by PCR using the primers BIGH3 F1: 5′-CTT TCC CAC ATG CCT CCT CGT-3′ (forward) and BIGH3 Rmut: 5′-TCT CAG GCC TCA GCT TCT CCC TGC-3′ (reverse). The “T” nucleotide at position 21 in the sequence of the reverse primer was replaced for a “C” to generate the PstI restriction site in the mutated allele. The 221 products were digested using the PstI restriction enzyme for 3 h at 37 °C. Fragments were resolved by electrophoresis in a 4% Nusieve agarose (3:1) gel, stained with ethidium bromide, and visualized under ultraviolet light. After digestion, the “T” allele consisted of three fragments of 21, 76, and 124 base pairs (bp), and the “C” allele consisted of two fragments of 97 and 124 bp.

## Results

### Clinical findings

The four-generation lattice corneal dystrophy type I family studied consisted of nine affected and nine unaffected individuals (Figure 1). Eight relatives of the proband were examined clinically. Five of them were affected by LCD type I (two males and three females) and three were not affected (two males and one female), as shown in the pedigree (Figure 1). The phenotypic features of affected and unaffected family members are summarized in Table 1. The age of onset of patients II-1, III-2, and IV-2 were obtained from an interview of patient II-1, although they were not clinically analyzed in this study. The proband was an eight-year-old boy (IV-5). The presence of LCD type I in three continuous generations indicates an autosomal dominant transmission. All patients clinically studied reported the beginning of lesions in a synchronic way in both eyes except for patient II-2 who noticed the lesion in the second eye one year after the lesion in the first eye. Visual sharpness ranged from 1.0 to 0.13; the worst were in the older generations. All patients had different degrees of astigmatism, with intraocular pressure (IOP) within the normal range (IOP<21) and had normal stereopsis.

#### Case II-3

**Figure 2:**
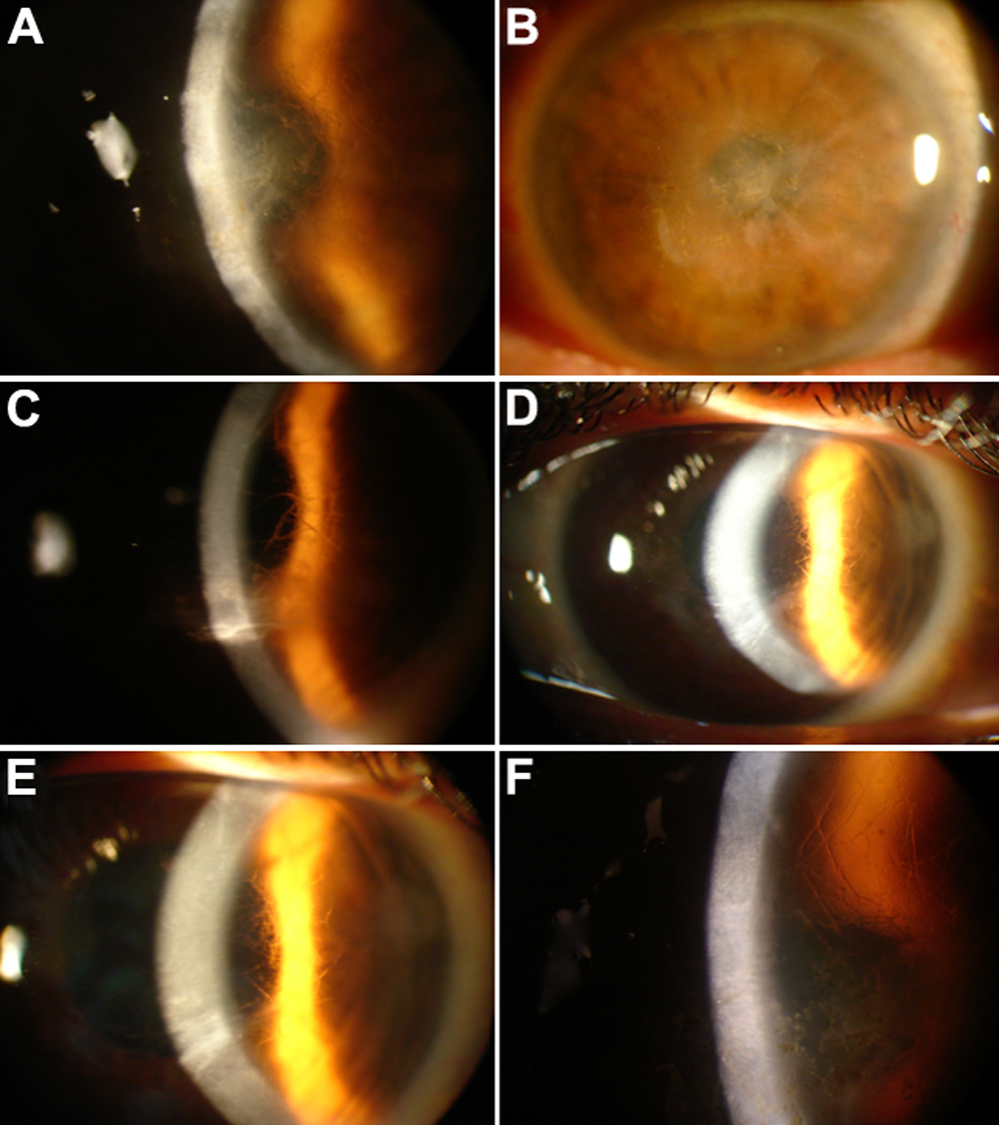
Corneal phenotype analyzed by slit lamp examination. Slit lamp photographs of patient II-3 at 69 years of age show an irregularity of the epithelial surface with subepithelial and anterior stromal scarring, resulting in diffuse clouding of the central cornea (**A** and **B**; OD and OS, respectively). The image of Patient III-5 at 42 years of age shows a network of linear opacities associated with other smaller opaque spots and refractile lattice lines (**C**). The photographs of case III-6 at 40 years of age show the appearance of corneal grafts (**D** and **E**). The left cornea contains opacifications from presumed recurrent disease (**D**). Fine branching lattice lines may be seen in the peripheral anterior stroma (**E**). The image of patient IV-5 at eight years of age shows the presence of large, ropy lattice lines in the anterior stroma. A clear area is preserved around the corneoscleral limbus (**F**).

The proband’s grandfather, at 69 years of age, was examined and diagnosed with LCD. His symptoms initiated with sensitivity to sunlight, episodes of ocular pain, and redness at 42 years of age. Slit-lamp examination revealed irregularity of the epithelial surface with subepithelial and anterior stromal scarring resulting in diffuse clouding of the central cornea (Figure 2A,B). Asymmetric commitment and vascularization of the cornea was observed. He was classified in maximal severity according to best-corrected vision (0.13 for both eyes) and corneal commitment. Typical, fine branching lattice lines could be seen in the peripheral anterior stroma, which was outside the area of central opacification. Central corneal sensation was significantly decreased, and the patient had a history of recurrent corneal erosions in the fourth and fifth decades of life.

#### Case III-5

The symptoms of the proband’s aunt began with episodes of acute ocular pain, redness, and photophobia at 30 years of age. The frequency and severity of these episodes increased coincident with a gradual deterioration of vision in both eyes. At 42 years of age, she was classified in maximal severity according to best-corrected vision (0.28 in the right eye [OD] and 0.13 in the left eye [OS]) and corneal commitment. Slit-lamp examination revealed an irregular epithelial surface with subepithelial and anterior stromal scarring, resulting in diffuse clouding of the central cornea. She showed a network of linear opacities associated with other smaller opaque spots and refractive lattice lines (Figure 2C). No vascularization of the cornea was observed. Central corneal sensation was decreased.

#### Case III-6

The proband’s mother, at 40 years of age, initially presented a mild subepithelial scarring and opacification, which gradually progressed to include elevated subepithelial opacities, fine lattice lines, diffuse “ground glass” haze in the anterior stroma, and corneal grafts (Figure 2D,E). Best-corrected vision was 0.5 OD and 0.4 OS. The left cornea contained opacifications from presumed recurrent disease. Fine branching lattice lines could be seen in the peripheral anterior stroma.

#### Cases IV-3 and IV-4

Although patients IV-3 and IV-4 did not have lesions at time of the exams, they had a history of recurrent corneal erosions in both eyes since the ages of three and four, respectively.

#### Case IV-5

The proband had a history of recurrent corneal erosions in both eyes, which began when he was four years old. Slit lamp examination showed the presence of large, typical fine branching lattice lines in the anterior stroma in OD (Figure 2F). A clear area was preserved around the corneal-scleral limbus. Best-corrected vision was 0.5 in the right eye (OD) and 0.67 in the left eye (OS).

The average age at onset for affected family members was 18.9±15.7 years old (3–42 years old; Table 1). However, the age of the beginning of symptoms has been progressively younger in succeeding generations. Generation II includes two individuals (II-1 and II-3) with the age of onset at 37 and 42 years old, respectively; generation III includes three individuals (III-2, III-5, and III-6) with the age of onset at 17, 28, and 30 years old, respectively; and generation IV includes four individuals (IV-2, IV-3, IV-4, and IV-5) with the age of onset at 5, 3, 4, and 4 years, respectively.

### Molecular genetic analyses

**Figure 3:**
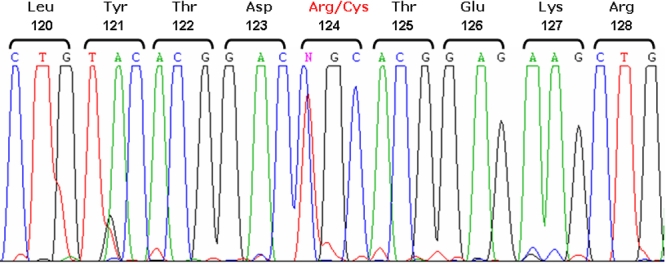
Detection of the pathogenic mutation (R124C) in the proband. The DNA sequence around codon 124 of the *TGFBI* gene is presented. The sequence in the proband shows a heterozygous, single-base C to T transition at nucleotide 417 (R124C) in the sense strand. The codon numbers are indicated at the top of the figure.

**Figure 4:**
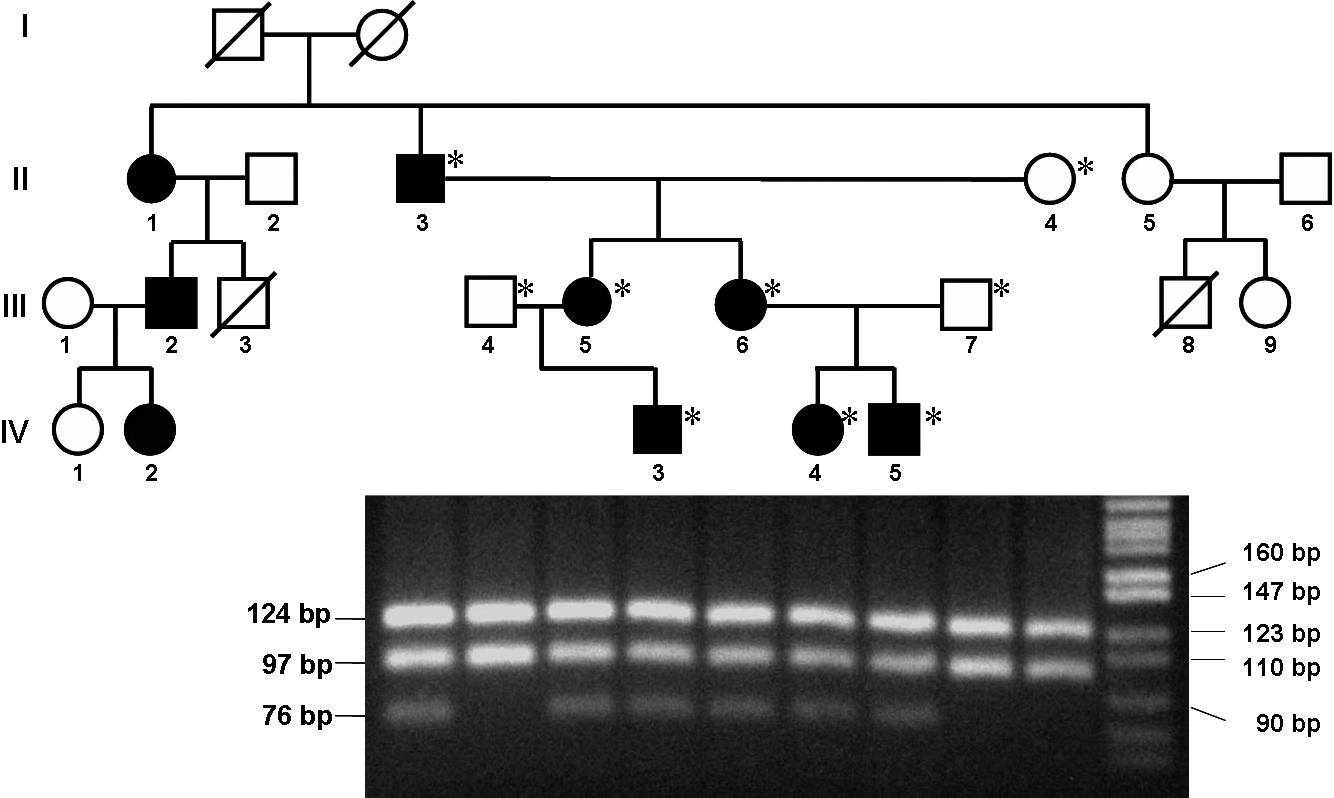
Analysis of R124C mutation by polymerase chain reaction-restriction fragment length polymorphism. All affected individuals carry the same mutation in exon 4 of the *TGFBI* gene. Exon 4 was amplified from each family member and digested with PstI. The products were analyzed by a 4% agarose gel electrophoresis along with a standard marker (MW) as indicated.

Exon 4 of *TGFB1* of the proband was analyzed by sequencing (Figure 3). The sequence revealed a heterozygous missense mutation, R124C (417C>T). To discriminate between normal and mutated alleles, we developed a new PCR-RFLP method. The mutation was analyzed in six affected (including the proband) and three healthy family members, revealing an identical heterozygous change in all affected. As expected, the mutation was absent in the unaffected individuals (Figure 4).

## Discussion

All affected family members showed phenotypic characteristics corresponding to LCDI. Within the family group, the patients showed a variable commitment that might be explained not only with the course of the variant disease but also with the amount of stromal damage over the years, which apparently increased with age.

We report a case with a heterozygous mutation detected by sequencing in exon 4 of *TGFBI*. This alteration has been reported previously in several ethnic groups [6,11-21]. The molecular analysis by the PCR-RFLP method developed here offers an easy, rapid, and accurate diagnosis of patients with R124C.

The genealogical information for the family allows us to trace the mutation only as far as the proband’s maternal grandfather who seems to be of Spanish origin. There is no information indicating that the great grandparents had any ophthalmic disorder, which suggests this is a new mutation.

We observed a decrease in the age of onset in three succeeding generations in the family of the proband. Analyzing previous reports of LCD1 families, we found one case describing the same point mutations in a family with decreasing age of diagnosis in successive generations similar to ours [25], although they do not document the age of onset. This could be similarly interpreted as genetic anticipation of the disease. Anticipation has been frequently described for diseases caused by the expansion of unstable triplet repeats, which is not the case with LCD1.

Anticipation has been also described for point mutations, insertions, and deletions of a small group of genes including *TCOF1*, located in 5q32 and mutated in Treacher Collins syndrome (TCS; OMIM 154500) [26]; *EBP*, a gene located in Xp11.22-p11.23 and mutated in X-chromosomal dominant chondrodysplasia syndrome (CHH, OMIM 302960) [27]; the transthyretin gene (*TTR*), located in 18q12.1 and mutated in familial amyloid polyneuropathy (FAP, OMIM:176300]) [28]; and copper–zinc superoxide dismutase 1 (*SOD1*), a gene located in 21q22.1 and mutated in amyotrophic lateral sclerosis (ALS, OMIM 105400). In some of these diseases, a genetic explanation of anticipation has been suggested. For example, in the chondrodysplasia syndrome, somatic mosaicism and differences in X chromosome inactivation can explain the phenomenon of anticipation and in amyloid polyneuropathy, the differences in the age of disease onset might be explained by genetic interactions among multiple loci.

In our case, genetic anticipation might be explained in at least three ways. One way is the fact that a previous ophthalmic history of the parents and grandparents probably prompted the family to look earlier for medical examination. It is possible that several mild cases of the syndrome, especially when the affected person does not feel impaired, will go undiagnosed until the occurrence of a more severely affected sibling or offspring. Another explanation of anticipation may be the result of the loss of a protective allele or another interacting gene from the affected parent. Thus the protective allele or interacting gene could ameliorate the effect of the mutated allele in the affected parent. Since such protective allele or protective gene could be absent in the offspring, it can not exhibit any protective effect in these subjects. The final possible way may be the gain of a susceptibility allele from the non-affected parent, which could worsen the effect of the mutated allele. If we consider that individuals III-4 and III-7 are not related, the probability of gaining a susceptibility allele simultaneously by individuals IV-3, IV-4, and IV-5 is low.

In conclusion, since the mutation at the hot spot nucleotide 417 can be easily, rapidly, and cost-effectively evaluated by PCR sequencing or by PCR-RFLP, identification of this mutation will allow Chilean patients to benefit from a timely and accurate molecular diagnosis of LCD type I. Genetic testing of *TGFBI* mutations in LCD type I patients will also contribute to improve their clinical classification, management, and eventual genetic counseling. Although the R124C mutation is one of the genetic causes of the disease, different genetic and environmental factors may govern the age of onset.
